# A New Web Score to Predict Health Status in Paediatric Patients with Chronic Diseases: Design and Development of the PENSAMI Study

**DOI:** 10.3390/children8121094

**Published:** 2021-11-26

**Authors:** Francesca Mastorci, Lamia Ait-Ali, Pierluigi Festa, Marco Martini, Luigi Gagliardi, Giovanni Calabri, Giancarlo La Marca, Gabriele Trivellini, Anselmo Casu, Stefano Dalmiani, Paolo Marcheschi, Simona Celi, Alessandro Pingitore

**Affiliations:** 1 Clinical Physiology Institute, Consiglio Nazionale delle Ricerche Area della Ricerca di Pisa (CNR), 56124 Pisa, Italy; mastorcif@ifc.cnr.it (F.M.); aitlamia@ifc.cnr.it (L.A.-A.); gabriele.trivellini@ifc.cnr.it (G.T.); webmaster@easywebmaster.eu (A.C.); 2 Fondazione G. Monasterio, Regione Toscana, 56226 Pisa, Italy; gigifesta@ftgm.it (P.F.); dalmiani@ftgm.it (S.D.); paolo.marcheschi@ftgm.it (P.M.); celi77@ftgm.it (S.C.); 3 Pediatric Allergology and Pulmonology, San Donato Hospital, 52100 Arezzo, Italy; marco.martini@uslsudest.toscana.it; 4 Pediatric Unit, Usl Nord Ovest Toscana, 55041 Viareggio, Italy; luigi.gagliardi@uslnordovest.toscana.it; 5 Unit of Pediatric Cardiology, Azienda Ospedaliero-Universitaria Meyer, 50139 Firenze, Italy; giovanni.calabri@meyer.it; 6 Newborn Screening, Clinical Chemistry and Pharmacology Lab Meyer Children’s Hospital, 50139 Firenze, Italy; giancarlo.lamarca@meyer.it

**Keywords:** comprehensive integrated health algorithm, ontology, chronic degenerative diseases, paediatric patients, quality of life, precision medicine

## Abstract

Paediatric chronic diseases (CD) are characterised by their ongoing duration and the fact that they are often managed throughout the lifespan, with the need to adjust lifestyle and expectations with the limitations coming from the CD. The aim of the PENSAMI study is to not only cure the disease, but to also care for the person from a clinical and psychosocial perspective. Data will be collected from 150 paediatric patients affected by heart disease, diabetes, and asthma admitted during in-hospital stay or outpatient visits, and from 200 healthy control subjects. The protocol will consist of two phases. The first one will aim at elaborating the predictive model by detecting (clinical, anthropometric at birth, environmental, lifestyle, social context, emotional state, and mental abilities) in order to develop a model predictive of the events considered: (1) re-hospitalisation; (2) severity and progression of the disease; (3) adherence to therapy; (4) HRQoL; (5) obesity and metabolic syndrome; (6) illness-stress related; (7) school drop-out; (8) school performance. The second one will address validating the previous predictive model. This model will aim to: (1) understand, prevent, and halt the progression of childhood CD; (2) develop new and improved diagnostic tools; (3) pave the way for innovative treatments and additional therapies to traditional clinical practice; and (4) create truly personalised therapeutic and preventive strategies in various sectors, such as cardiology, diabetes, and respiratory diseases.

## 1. Introduction

Chronic diseases are characterised by their ongoing duration and the fact that they are often managed throughout the lifespan [1]. This changes a patient’s lifespan and generates the need to adapt and develop an understanding of the relationship between the demands of life and those of the condition [2]. In this context, the spectrum of risk factors ranges from purely genetic to behavioural, psychosocial, and environmental ones, creating an integrated and complex causal pathway to consider when applying preventive strategies to improve health and well-being. In this process of evolution, different disease conditions share common pathophysiological mechanisms, such as the activation of hormonal, inflammatory, and nervous systems that are initially protective, but become potentially harmful when continuously activated [3]. In particular, these patients experience a range of medical needs which can alter their daily family routines and activities, and the whole family must adjust to a new way of life in order to deal with the day-to-day management of the condition [4]. This lifestyle change can be the cause of significant and persistent stress, particularly in young people, who deal with the complex developmental tasks associated with their age in addition to the disease, learning to manage their own illness as they prepare for the transition to adulthood [5,6]. Among chronic diseases in the paediatric population, asthma, diabetes, and congenital heart disease have about an estimation of 10–18% [7,8,9,10]. Advances in treatments, and the effective possibilities of the control of chronic-degenerative and congenital diseases have resulted in longer lifetimes for people afflicted, but on the other side, they require daily self-management throughout their life span, with restricted physical and social activities [1]. Consequently, there has been a call for new outcome measures that reflect a more holistic approach to management, introducing the concept of health-related quality of life as a result of the relationship between mind and body, and the link between physical and psychological health. Therefore, the efficacy of any intervention is not only estimated in terms of mortality reduction, but also in terms of quality of life, reduction of disease progression, and also considering other daily-life events. Health-related quality-of-life (HRQoL) measurement provides a comprehensive description of an individual’s health, even in the presence of a disease, representing a multidimensional construct including physical, psychological, and social well-being and functioning. Several key ideas define the concept of HRQoL in children and adolescents, in healthy or illness conditions. First, is the idea that individuals have their own unique perspective on HRQoL, which depends on present lifestyle, past experiences, hopes for the future, dreams, and ambition. Secondly, when used in a medical context, it follows from the widely accepted definition of health put forward by the World Health Organisation as the state of complete physical, mental, and social wellbeing, and not merely the absence of disease or infirmity. According to this perspective, the aspects comprising HRQoL in chronically ill paediatric patients can be shared into two factors: disease-related factors (age of onset, disease severity, complications, treatment, sense of normality and positive attitude towards the disease), and non-disease-related factors (age, gender, socioeconomic status, support of parents, social wellbeing, and support) [11]. Recent literature has highlighted that psychosocial variables can assume a crucial role, more than the presence, per se, of the physical and clinical dimensions of a chronic condition [12,13,14]. Supporting this idea, clinical settings have progressively recognised the need to complement traditional health indicators with psychosocial factors [15,16,17]. Therefore, the inclusion of variables associated with health-related quality of life within a multidimensional approach in clinical decision making is useful for both managing symptoms and improving psychosocial care [18,19,20]. To solve this problem, a new framework is urgently needed to manage not only clinical parameters, but also the impact that the disease may have on the socialisation process, emotional health, and general limitations in ordinary activities, with particular focus to the paediatric context [21,22].

According to the WHO objectives for chronic disease prevention, the development of an integrated approach that will target all major common risk factors of chronic diseases is the most cost-effective way to prevent and control them. An integrated approach responds not only to the need for intervention in major common risk factors with the aim of reducing premature mortality and morbidity of chronic diseases, but also the need to integrate primary, secondary, and tertiary prevention, health promotion, and related programmes across sectors and different fields. Two critical events are converging that provide the basis for an immediate and evolving need in the management of chronic disease in children and young people. The number of children and young people with chronic disease has increased worldwide over recent decades, estimating that 10% of adolescents suffer from a chronic disease [23]. Furthermore, technology is enabling real-time monitoring and reporting of measureable activities and events. In this framework, the new era of computer technology is actually pervasive in healthcare, and, in particular, web-tools provide the opportunity to gather data in a fast, cheap, and low time-consuming way, favouring also the possibility to integrate information with each other. It is critical, therefore, to consider how to involve the subject in the process of monitoring and improving/maintaining their health and behaviours to reduce outcomes and overall costs, and improve quality of life. The integration of data, both clinical and linked to psychosocial dimensions proposed here, could offer potential by improving disease prevention, patient outcomes and compliance, and personalised therapeutic strategies. The general objective of the PENSAMI project, “A Precision mEdiciNe-baSed frAMework to pediatric patients with chronic dIseases”, is to detect variables, belonging to clinical, anthropometric measures at birth, environment, lifestyle, social context, emotional state, and mental abilities, that have an impact on HRQoL. Once these variables are defined, a healthy index will be created, and the effectiveness of this index will be tested in a large and multicentre clinical study. The healthy index obtained will be used to predict the considered events: (1) re-hospitalization; (2) severity and progression of the disease; (3) adherence to therapy; (4) HRQoL; (5) obesity and metabolic syndrome; (6) stress-related illness; (7) school drop-out; (8) school performance.

## 2. Materials and Methods

### 2.1. Approach of the Presented Protocol

**Figure 1:**
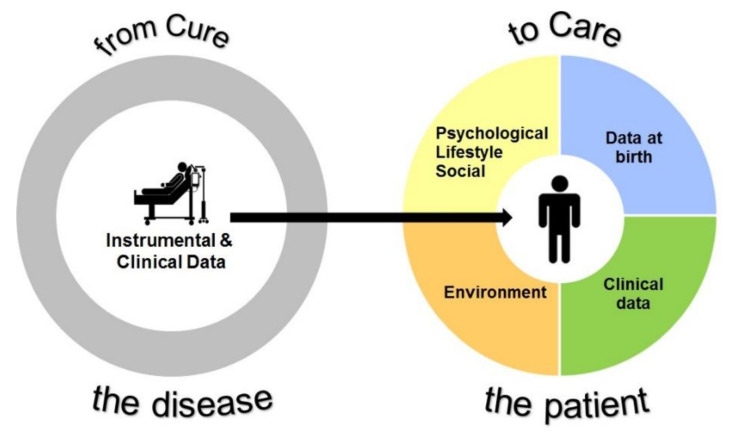
The PENSAMI framework.

The PENSAMI project is a large-scale prospective multicentre study that focuses on secondary prevention of paediatric patients with chronic diseases. The PENSAMI approach is based on a perspective that an integrated and multidimensional health framework, including clinical, lifestyle, psychosocial, and environmental determinants, is effective in more accurately stratifying patients with a higher probability of events, and improving the quality of life of paediatric patients with chronic diseases. This point of view is based on the concept that illness status has a significant impact on the personal characteristics of subjects, such as emotional status, social relationships, and cognitive skills, that, in turn, may worsen clinics. Therefore, the PENSAMI concept is in line with the definition of precision medicine, that is an emerging approach for prevention, diagnosis, and cure of diseases that takes into account subjective changes in genetic, environmental, and lifestyle assets. In this view, therapeutic and preventive strategies will be tailored to individuals or groups, rather than using a one-size-fits-all approach in which everyone receives the same care (Figure 1).

The system design process will start with the development of ontologies based on different, but functionally complementary, perspectives in the knowledge domain of paediatric chronic diseases. The domains considered will be clinical data, anthropometric data at birth, environmental data, and on lifestyle habits, social context, emotional status, and mental skills. At the follow up, one year after enrolment, the events measured will be re-hospitalization, severity and progression of the disease, adherence to therapy, health-related quality of life, obesity and metabolic syndrome, stress-related illness, school drop-out, and school performance, in order to better define the knowledge domain, and to cover the predictive model.

### 2.2. Participants

We will recruit about 150 paediatric patients equally distributed by sex into the three pathologies: congenital heart disease; diabetes; and asthma; in the age range between 9 and 16 years.

Clinical data, including clinical history, bio-humoral, and instrumental data, will be included in a database connected to the PENSAMI platform. Participants will be included if they: (1) have been diagnosed with a specific chronic disease (diabetes mellitus, asthma, and congenital or acquired chronic heart disease); (2) are aged between 9 to 18 years; (3) have cognitive skills that allow them to fill the questionnaire autonomously. The control group will consist of about 200 healthy adolescents of the same age enrolled in middle school. All procedures performed in the study were in accordance with the ethical standards of the institutional and/or national research committee, and with the 1964 Declaration of Helsinki and its later amendments or comparable ethical standards. The protocol was approved by the Regional Ethics Committee for Clinical Trials of the Tuscany Region—Section Paediatric Ethics Committee—Azienda Ospedaliero Universitaria Meyer (36/2021). All parents or legal guardians gave informed consent and authorised the researchers to use their data in accordance with Italian law, and were free at any stage to withdraw their consent.

### 2.3. Clinical Study Design

**Figure 2:**
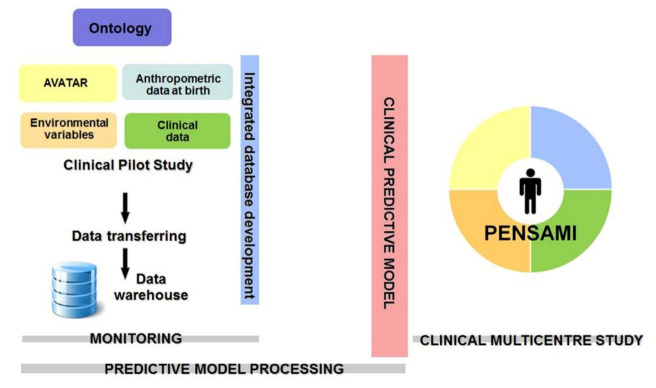
Overall study design.

The clinical study will consist of two different steps. The first phase will be aimed at elaborating the predictive model through the detection of variables (clinical, anthropometric measures at birth, environmental parameters, lifestyle, social context, emotional state, and mental abilities) that have an independent impact on HRQoL and on the other events considered: (1) re-hospitalization; (2) severity and progression of the disease; (3) adherence to therapy; (4) HRQoL; (5) obesity and metabolic syndrome; (6) stress-related illness; (7) school drop-out; (8) school performance; in order to develop a predictive model of the considered events. The basic idea will be to analyse the collected data by using specific machine learning approaches based on random forest (RF) algorithms. In order to guarantee the expected results, several sub-steps of RF will be performed. Moreover, the robustness of the pilot study will be guaranteed by the final double-correlation process: each output variable will be processed with respect to the input variables and with respect to the other output variables to define even potential mutual correlation between the PENSAMI final scores. The second phase will be targeted at validating the predictive model that will replicate on a large scale with a multicentre study that was obtained in the previous phase. The schematic workflow of the clinical study is reported in Figure 2.

For clinical and control groups, first and second phase (predictive model processing and multicentric clinical study) will consist of two steps. The enrolment step (in hospital and at school for controls) consists of: (i) clinical evaluation; (ii) collection of lifestyles, social context, emotional state, and mental skills by means of a web-platform, through self-administration of dedicated psychometric tests [24,25]; (iii) environmental data (e.g., air pollution, electromagnetic fields, etc.) and anthropometric measures at birth. The follow-up step (1 year from enrolment) consists of: (i) clinical evaluation to define the clinical status and other events considered; (ii) evaluation of clinical and non-clinical events ((1) re-hospitalization, (2) severity and progression of disease, (3) adherence to therapy, (4) HRQoL, (5) obesity and metabolic syndrome, (6) stress-related illness, (7) school drop-out, (8) school performance); (iii) lifestyles, social context, emotional state, and mental skills information.

### 2.4. Ontology and Data Analysis

The PENSAMI project will develop the PENSAMI web-tool to gather data from the already existing platform (C7 for clinical data; ARPAT platform for environmental data; regional platform for anthropometric data at birth). The creation of this platform will provide the opportunity to gather data in a fast, cheap, and low time-consuming way, favouring the opportunity to integrate the information with each other. The clinical data will include general and specific parameters for each disease. All patients with chronic illness will be characterised with a minimum dataset: age at the diagnosis; number of hospitalization last year; number of day services/day hospitals in the last year; number of outpatient visits in the last year; limitation of activities.

For the different diseases, the variables that will be considered are:

Heart disease:
  - Diagnosis score severity;
  - Number of interventions;
  - Correction vs. palliative repair (for congenital heart disease);
  - Residual defect;
  - Cyanosis;
  - Peripheral O_2_ saturation;
  - Functional class;
  - Shortness of breath;
  - Congestive therapy;
  - Arrhythmic therapy;
  - Coagulation/platelet therapy.
Asthma:
  - Disease severity;
  - Night time symptoms;
  - Daytime symptoms;
  - Rescue bronchodilator use;
  - Airway narrowing;
  - Asthma control test.
Diabetes:
  - Glycaemic control;
  - Number of daily injection;
  - Nutritional models;
  - Hypoglycaemia/Hyperglycaemia episodes;
  - Severity score.
With the neonatal screening program, it is possible to have the following data available:
  - Birth weight;
  - Gestation weeks;
  - Type of birth;
  - Phenylketonuria and other metabolic illnesses;
  - Biotin deficiency;
  - Congenital hyperthyroidism;
  - Galactosemia;
  - Cystic fibrosis;
  - Lysosomal storage diseases (LSD);
  - Severe combined immune deficiency.

For lifestyle habits, considering also the social context, emotional state, and mental skills, the variables will be grouped into 10 dimensions: physical well-being; psychological well-being; moods and emotions; self-perception; autonomy; parent relations and home life; financial resources; social support and peers; school environment; and social acceptance.

Environmental data will consist of:
  - air pollution data;
  - electromagnetic fields;
  - radioactivity;
  - products in the waters;
  - noise.

**Figure 3:**
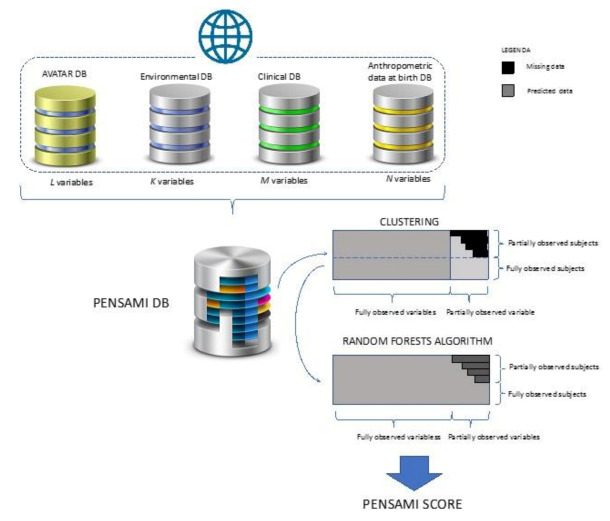
PENSAMI platform ontology.

With the exception of anthropometric data at birth, which will be acquired at the time of enrollment, all other data will be obtained both at enrollment and at follow-up. All parameters will be monitored in all subjects (patients and controls), except obviously the clinical parameters, which are specific for the different categories of patients. Figure 3 depicts the overall schema of our platform. Data from the four databases (clinical and environmental data, regional platform for anthropometric data at birth, and HRQoL data obtained from AVATAR web platform) will be collected in our specific database (Pensami DB).

Based on an initial analysis of database structure and related content, a controlled term vocabulary will be compiled, containing a formal definition of the semantic content for each vocabulary item. A clinical supervisor will be responsible for the content, and will ensure a coherent approach among different clinical specialties. Since missing data are common in clinical and public health studies, according to previous studies, a dedicated machine learning algorithm based on random forest (RF) will be used [26]. Firstly, the collected variables will be processed with a clustering, then the RF algorithm will be applied. The choice of the RF algorithm is due to its characteristic that it can: (1) handle mixed types of missing data; (2) address interactions and potential nonlinearity; (3) scale to high-dimensions while avoiding overfitting; and (4) yield measures of variable importance useful for variable selection [27]. The multiple imputation method, which involves creating multiple complete versions of data with missing values imputed through random draws from distributions inferred from the observed data, will be associated with random forest analysis to address the missing data issue in an effective way.

## 3. Discussion

In this study, we presented a new integrated approach aimed not only at curing the disease, but also at caring for the person, in particular, during critical time windows, such as childhood and adolescence. According to this perspective, the development of an integrated predictive model has the potential practical impacts of: (1) improving stratification of the patient population, thus, identifying the patients at higher risk of events; (2) taking care of the patients by looking at subjective aspects that could be negatively influenced by their illness, which, in turn, could worsen their own clinics; (3) improving HRQoL through improved therapeutic management, including the adoption of personalised intervention strategies that adopt a complementary and holistic approach, beyond pure pharmacological methods. The literature review has highlighted the need for solutions to enhance diagnosis, and interactive technologies have shown a huge potential within the specific framework of health promotion, intended as the promotion of disease awareness, self-management and treatment, and health behavioural change and healthy lifestyles promotion [28]. Within the e-Health topic, advancing digitization goes along with great potential in health care, however, there is a lack of cohesive evaluation standards among the large number of mobile apps/web-tools currently available. The PENSAMI score described here could have a positive impact on the quality of care regarding diagnosis, and support medical education programs, promoting better patient care and better, quicker, and more confident clinical decision processes by physicians. The PENSAMI project meets the main aim in the field of personalizing health and care: to improve the public health and the general well-being of individuals and society. This is of increasing importance because of the aging of the global population, and their progression of chronic diseases that require expensive management and can negatively impact quality of life. Progress in surgical and medical therapy has enabled an improvement of survival in patients with chronic disease in paediatric age groups. Associated with this, is the concept of health-related quality of life: a functional outcome reflecting a patient’s reported health status, which can be substantially worse in patients with chronic disease [29,30]. According to previously published reports, more psychosocial elements became determinants in HRQoL than clinical factors [31]. In addition, there is scientific evidence that poor HRQoL is a negative predictor of disease progression [32,33]. Because of this, the PENSAMI project aims to improve individual self-control of health and disease prevention through the development of a DSS based on predictive modelling and data obtained from clinical data, anthropometric data at birth, environmental parameters, lifestyle habits, social context, emotional status, and mental abilities. The development of a clinical predictive model will support the design of a web-tool for the improvement of the adolescent’s well-being through a dynamic and personalised interface, which will provide immediate automated feedback that will allow doctors and other stakeholders (school, family, etc.) to monitor progress. From a public health point of view, the development and implementation of this integrative approach from proof of concept to confirmation of clinical efficacy will be a real step forward in individual awareness and the empowerment of health promotion and disease prevention. Evaluating these parameters in clinical practice would be time-consuming and would prohibitively increase the cost of primary prevention. However, the approach of the PENSAMI project aims to prove that this can be done easily and with limited costs in the new era of smart devices.

## 4. Conclusions

The PENSAMI project will produce an advanced integrated approach to paediatric patients with chronic disease, especially for the development of a personalised model that is based on a set of clinical parameters, and variables that are most affected by individual variability and, at the same time, more influenced by the environment to which the subject is exposed. This integration by computer modelling and information fusion will, on the one hand, allow for the personalised, quantitative, and real-time assessment of psychosocial profile and clinical status, and, on the other hand, will offer the potential to improve disease prevention, as well as patient outcomes and compliance, while lowering the cost of healthcare, and taking advantage of currently available technologies. Predictive model processing will have inherent technological innovative features, related to: (i) the development of a PENSAMI web-tool, highly flexible and adaptable in its potential applications; (ii) the implementation of an intelligent system able to efficiently and effectively exploit different information provided by the integrated data; (iii) the improvement of user-centric applications able to provide a friendly user interaction which allows the user to develop personalised strategies to behavioural changes. Although at the time of the study, it was still difficult to perceive the true impact of tools on patient health outcomes, the PENSAMI score will have three important potential clinical implications: (1) the improvement of disease management and prevention; (2) the improvement of patient outcomes and compliance; (3) the optimization of therapeutic strategies through a personalised approach. PENSAMI could become a powerful tool to reduce clinical and non-clinical events by a combination of approaches, such as refinement of risk prediction, guideline compliance, and improvement in adherence, including environmental and social interventions.

## Data Availability

Not applicable.
